# Exosomal miRNAs as potential biomarkers for insulin resistance and metabolic dysfunction-associated steatotic liver disease in children with obesity

**DOI:** 10.3389/fendo.2025.1683403

**Published:** 2025-11-18

**Authors:** Eu-Seon Noh, Yoonseo Yeum, Hye Young Jin, Seongho Ryu, Il Tae Hwang

**Affiliations:** 1 Department of Pediatrics, Kangdong Sacred Heart Hospital, Seoul, Republic of Korea; 2 Department of Integrated Biomedical Science, Soonchunhyang University, College of Medicine, Cheonan, Republic of Korea; 3 Soonchunhyang Institute of Med-Bio Sciences (SIMS), Cheonan, Republic of Korea; 4 Department of Pathology, College of Medicine Soonchunhyang University, Cheonan, Republic of Korea

**Keywords:** microRNA, metabolic dysfunction-associated steatotic liver disease, insulinresistance, metabolic dysregulation, TyG-ALT index

## Abstract

**Background:**

MicroRNAs (miRNAs) have emerged as potential biomarkers for insulin resistance-related conditions such as obesity, metabolic syndrome, and metabolic dysfunction-associated steatotic liver disease (MASLD) in adults. However, data in pediatric populations remain limited. This study aimed to compare serum exosomal miRNA expression among children with both obesity and MASLD, children with obesity without MASLD, and healthy controls, and to evaluate associations with insulin resistance markers.

**Methods:**

Thirty prepubertal children (5 males and 5 females per group) were enrolled: children with both obesity and MASLD (n=10), children with obesity without MASLD (n=10), and healthy controls (n=10). Serum exosomal miRNAs were analyzed using next-generation sequencing. Differential expression analyses were performed between groups, and correlations with clinical and metabolic parameters were assessed, including the triglyceride-glucose (TyG) index, TyG index adjusted for ALT (TyG-ALT), and the homeostatic model assessment for insulin resistance (HOMA-IR).

**Results:**

All participants were prepubertal, with no significant differences in mean age across groups. miR-34a-5p, miR-122-5p, miR-885-5p, and miR-885-3p were significantly upregulated in children with both obesity and MASLD compared to both other groups, and positively correlated with AST and uric acid levels. Additionally, miR-122-5p, miR-885-5p, and miR-885-3p were associated with ALT and TyG-ALT index. Conversely, miR-570-3p and miR-32-3p were significantly downregulated in children with both obesity and MASLD and negatively correlated with AST, ALT, and TyG-ALT levels.

**Conclusions:**

Six exosomal miRNAs (miR-34a-5p, miR-122-5p, miR-885-5p, miR-885-3p, miR-570-3p, and miR-32-3p) were differentially expressed in children with both obesity and MASLD and showed strong correlations with markers of liver function and insulin resistance. These findings suggest their potential as non-invasive biomarkers for early identification of MASLD and related metabolic disturbances in pediatric populations.

## 1 Introduction

MicroRNAs (miRNAs) are small, single-stranded, non-coding RNA molecules, typically 21 to 23 nucleotides in length, which have emerged as key regulators of gene expression in various biological processes (1). Through precise post-transcriptional modulation, miRNAs play a significant role in maintaining cellular function and homeostasis (2). Dysregulation of miRNAs can have widespread effects on tissues and organs, including adipose tissue, the pancreas, liver, and muscle. Such alterations are often associated with metabolic disturbances, particularly obesity and related disorders (3–5).

Metabolic dysfunction-associated steatotic liver disease (MASLD) is the most common chronic liver disease in children and adolescents (6, 7). It ranges from simple hepatic steatosis to non-alcoholic steatohepatitis, fibrosis, and cirrhosis. Pediatric MASLD often presents with more severe inflammation and metabolic complications than adult-onset MASLD, making early detection particularly important (8, 9). In Korea, its relevance is growing with the increasing prevalence of pediatric obesity (10). In recent years, miRNAs have been studied as potential biomarkers of MASLD in children. Some studies have reported an association between liver enzymes, insulin resistance, and fat accumulation (11, 12). However, many of these studies focused on a limited number of miRNAs or did not clearly compare different clinical phenotypes.

In this study, we aimed to identify exosomal miRNAs associated with MASLD in children with obesity. By comparing miRNA profiles among children with obesity and MASLD, children with obesity without MASLD, and healthy controls, we aimed to explore miRNA expression patterns specifically associated with MASLD, beyond the effects of obesity alone. Associations between the selected miRNAs and surrogate markers of insulin resistance were also examined to better understand their potential roles in early metabolic dysfunction. These findings support the development of miRNA-based, non-invasive biomarkers for the early detection and risk assessment of pediatric MASLD.

## 2 Patients and methods

### 2.1 Patients

This study included three groups: children with obesity and MASLD (n = 10), children with obesity without MASLD (n = 10), and healthy controls (n = 10). Children with obesity were defined as those with a BMI at or above the 95th percentile. MASLD was defined as a US-FLI score of ≥ 2, while non-MASLD participants had a US-FLI score of < 2 (13). Healthy controls had a BMI below the 85th percentile, with no abnormalities detected on ultrasound or in routine blood tests. This study was approved by the Institutional Review Board of Kangdong Sacred Heart Hospital (IRB number: KDH 2022-11-008).

### 2.2 Clinical and laboratory assessments

The clinical parameters assessed included Tanner stage, growth velocity, bone age, and BMI. Laboratory tests were conducted on samples collected in the fasting state, and serum glucose, total cholesterol, HDL cholesterol, triglyceride, insulin, AST, and ALT levels were measured. An additional 4 mL of sample was collected in SST tubes for miRNA analysis.

### 2.3 Differential miRNA expression analysis

Blood samples were collected, and exosomal miRNAs were isolated using ExoQuick. RNA extraction and library preparation were performed with the QIAseq miRNA Library Kit (Qiagen) according to the manufacturer’s protocol. Sequencing was carried out on the Ion Torrent system (Ion 550 Kit-Chef, Thermo Fisher Scientific), yielding approximately 10–19 million reads per sample, with more than 90% of bases above Q20 and mapping rates of 50–75%, confirming sufficient data quality. Differential expression analysis was performed using edgeR (Bioconductor v3.38.4), and miRNAs with |log_2_ fold change| > 1 and p < 0.05 were considered statistically significant.

### 2.4 Statistical analysis

Statistical analyses were conducted using the SPSS Statistics software (version 28). ANOVA and Mann–Whitney U tests were used to compare data across the three groups. Spearman’s correlation analysis was performed to assess the correlations between six significant miRNAs and clinical parameters. Statistical significance was set at p < 0.05.

## 3 Results

### 3.1 Comparison of baseline and clinical characteristics

**Table 1** compares the baseline and clinical characteristics of the three groups: children with obesity and MASLD, children with obesity without MASLD, and healthy controls. All participants were approximately 8 years old. As expected, the weight SDS and BMI SDS were significantly higher in children with obesity—both with and without MASLD —compared to healthy controls. Liver function markers, including AST and ALT, and uric acid, were elevated in children with both obesity and MASLD. Additionally, insulin resistance markers, such as triglyceride glucose (TyG)-ALT and TyG-BMI indices, were significantly higher in children with both obesity and MASLD, indicating a greater degree of metabolic dysregulation associated with fatty liver disease.

**Table 1:** Baseline and clinical characteristics of the study groups: healthy controls, children with obesity without MASLD, and children with both obesity and MASLD.

	Obesity with MASLD	Obesity without MASLD	Control	P-value
Age (years)	8.66 ± 1.0	8.59 ± 1.04	8.25 ± 1.38	0.745
Height SDS	1.13 ± 1.21	1.02 ± 1.14	-0.19 ± 1.19	0.033
Weight SDS	2.52 ± 0.99	2.21 ± 0.49	-0.56 ± 0.55	<0.001
BMI SDS	2.91 ± 0.97	2.44 ± 0.40	-0.69 ± 0.59	<0.001
AST (IU/L)	55.4 ± 32.4	28.0 ± 8.40	29.9 ± 4.88	0.006
ALT (IU/L)	93.1 ± 82.8	19.3 ± 6.99	14.1 ± 3.72	0.001
Fasting glucose (mg/dL)	94.4 ± 6.70	88.4 ± 6.31	93.6 ± 5.62	0.083
Uric acid (mg/dL)	6.14 ± 0.85	5.06 ± 0.92	4.65± 0.60	0.001
Insulin (uU/mL)	10.4 ± 4.1	12.0 ± 4.06*	6.1 ± 2.8	0.009
HbA1c (%)	5.38 ± 0.23	5.35 ± 0.19	5.08± 0.21	0.045
Total cholesterol (mg/dL)	182.1 ± 20.70	183.9 ± 28.14	178.2 ± 20.36	0.857
HDL (mg/dL)	50.7 ± 6.80	53.5 ± 14.78	67.2 ± 8.75	0.004
LDL (mg/dL)	115.2 ± 15.53	115.0 ± 22.24	99.0 ± 19.9	0.121
Triglyceride (mg/dL)	101.7 ± 43.47 *	90.80 ± 28.58	60.2 ± 17.7	0.019
HOMA-IR	2.46 ± 1.04	2.64 ± 1.04 *	1.40 ± 0.68	0.027
TyG index	8.39 ± 0.44 *	8.25 ± 0.30	7.90 ± 0.30	0.015
TyG-ALT index	790.3 ± 709.4	159.16 ± 58.42	111.75 ± 31.10	0.001
TyG-BMI index	217.98 ± 38.36	180.42 ± 47.41	124.68 ± 12.06	<0.001
Elastography (kPa)	4.19 ± 1.20	3.35 ± 0.37	3.96 ± 0.43	0.069
US-FLI (score)	3.6	0	0	

*Post-hoc analysis showed a significant difference compared with the control group.

SDS, standard deviation score; TyG, triglyceride glucose index; HOMA-IR, homeostasis model assessment of insulin resistance.

### 3.2 Differential miRNA expression

**Figure 1:**
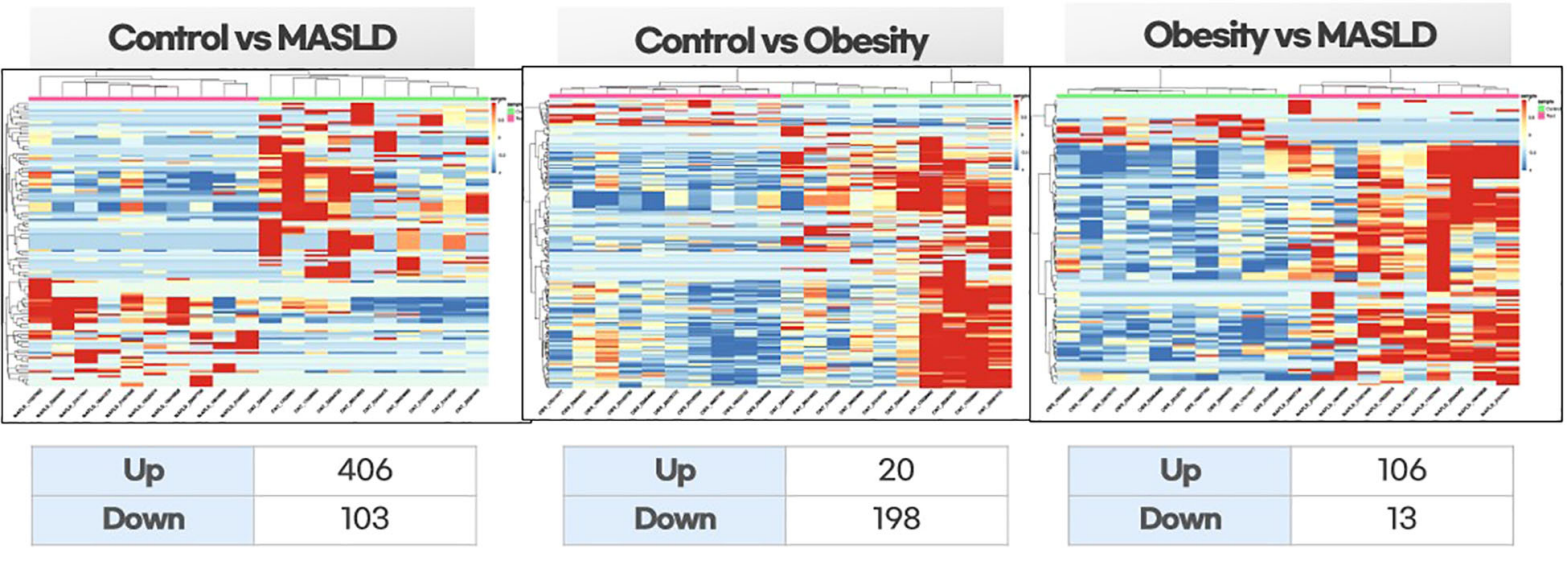
Differential expression heatmaps of exosomal miRNAs among the study groups: healthy controls vs. children with MASLD, healthy controls vs. children with obesity, and children with obesity vs. children with MASLD. Hierarchical clustering was performed on normalized expression data (z-scores). Red indicates high expression, and blue indicates low expression.

The differential expression of miRNAs across the three groups is shown in the heat map in **Figure 1**. **Figure 1A** shows a comparison between the control and children with both obesity and MASLD. There was a clear distinction in miRNA expression patterns, with approximately 406 upregulated and 103 downregulated miRNAs in children with both obesity and MASLD compared with the control group. **Figure 1B** shows a comparison between healthy controls and children with obesity without MASLD. In this comparison, 20 miRNAs were upregulated and 198 were downregulated in children with obesity compared to healthy controls. **Figure 1C** shows a comparison between children with obesity without MASLD and those with both obesity and MASLD. Children with both obesity and MASLD exhibited greater miRNA expression changes, with approximately 106 miRNAs upregulated compared to children with obesity without MASLD.

### 3.3 Key miRNAs associated with MASLD

**Figure 2:**
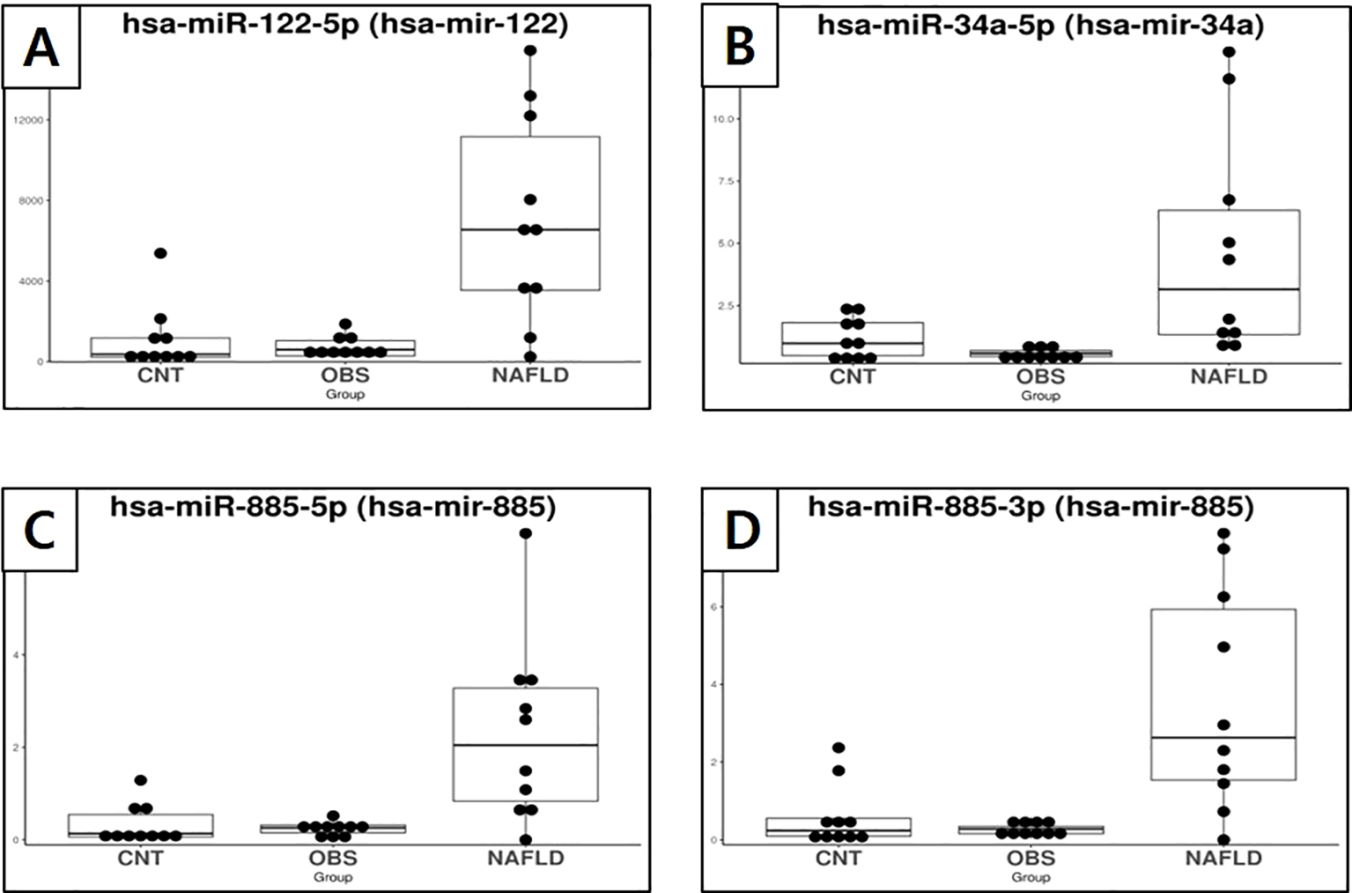
Box plots showing the expression of exosomal miRNAs in healthy controls, children with obesity without MASLD, and children with both obesity and MASLD, highlighting miRNAs that were significantly upregulated only in the latter group. **(A)** miR-122-5p, **(B)** miR-34a-5p, **(C)** miR-885-5p, **(D)** miR-885-3p.

**Figure 3:**
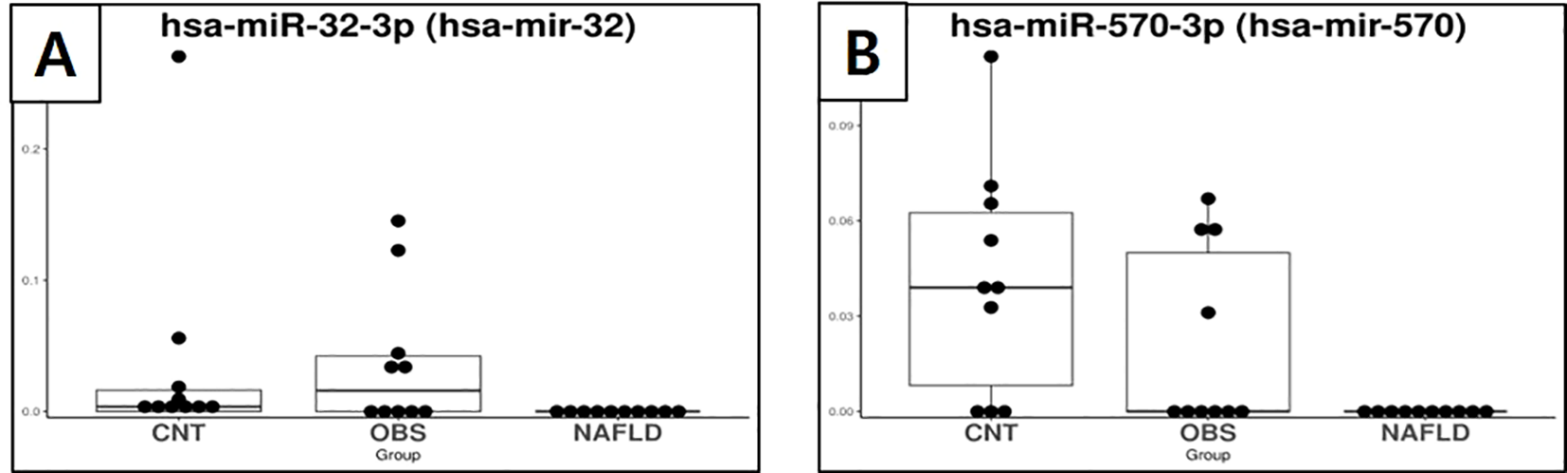
Box plots showing the expression of exosomal miRNAs in healthy controls, children with obesity without MASLD, and children with both obesity and MASLD, highlighting miRNAs that were significantly downregulated only in the latter group. **(A)** miR-32-3p, **(B)** miR-570-3p. miRNA, microRNA; MASLD, metabolic dysfunction-associated steatotic liver disease; CNT, control group; OBS, children with obesity without MASLD; ON, children with both obesity and MASLD.

Specific miRNAs that were significantly dysregulated in children with both obesity and MASLD compared with both the control and children with obesity without MASLD are shown in **Figures 2**, **3**. Box plots for each miRNA illustrate the log fold-change (log2FC) and statistical significance (p-value) for comparisons across the groups, confirming that these miRNAs were uniquely elevated in the children with both obesity and MASLD.

Specifically, miR-34a-5p (**Figure 2B**) had a log2FC of 3.29 and p-value of <0.001, indicating a significant increase in the children with both obesity and MASLD. Similarly, miR-122-5p (**Figure 2A**) had a log2FC of 2.34 with p-value < 0.001, whereas miR-885-5p (**Figure 2C**) showed a log2FC of 3.43 with a p-value < 0.001. Additionally, miR-885-3p (**Figure 2D**) was upregulated in the children with both obesity and MASLD (log2FC = 3.23, p < 0.001). In contrast, miR-32-3p (**Figure 3A**) and miR-570-3p (**Figure 3B**) were significantly downregulated in the children with both obesity and MASLD, with log2FC values of -3.31 (p = 0.03) and -3.86 (p = 0.02), respectively. Altogether, six candidate miRNAs, miR-34a-5p, miR-122-5p, miR-885-5p, miR-885-3p, miR-32-3p, and miR-570-3p, showed expression differences specific to the children with both obesity and MASLD.

### 3.4 Correlation analysis of miRNAs with clinical and metabolic parameters

**Table 2** shows the results of the correlation analysis between the six candidate miRNAs and various clinical and metabolic parameters. Notably, miR-34a-5p was positively correlated with uric acid levels (r = 0.417, p < 0.05). miR-122-5p, miR-885-5p, and miR-885-3p were positively correlated with ALT, and uric acid levels and the TyG-ALT index, with correlation coefficients ranging from 0.436 to 0.576 (p < 0.05). Conversely, miR-570-3p was negatively correlated with ALT (r = -0.499, p < 0.05) and both the TyG-ALT (r = -0.505, p < 0.05) and TyG-BMI (r = -0.556, p < 0.05) indices.

**Table 2:** Significant correlations (r, p < 0.05) between selected exosomal miRNAs and clinical/metabolic parameters in children with obesity and MASLD.

	miR-34a-5p	miR-122-5p	miR-885-5p	miR-885-3p	miR- 32-3p	miR-570-3p
Weight SDS	NS	**0.377**	NS	NS	NS	**-0.449**
BMI SDS	NS	NS	NS	NS	NS	**-0.567**
AST	**0.453**	**0.596**	**0.576**	**0.535**	NS	**-0.38**
ALT	NS	**0.576**	**0.574**	**0.461**	NS	**-0.521**
Uric acid	**0.597**	**0.647**	**0.589**	**0.635**	NS	NS
HbA1c	NS	NS	NS	NS	**-0.397**	NS
TyG-ALT	NS	**0.576**	**0.582**	**0.475**	NS	**-0.505**
TyG-BMI	NS	**0.386**	NS	NS	NS	**-0.399**
Elastography	NS	NS	NS	NS	**-0.383**	NS

Values represent Pearson correlation coefficients (r). Only significant correlations (p < 0.05) are shown.

NS, not significant; SDS, standard deviation score; TyG, triglyceride glucose index.

Bold values indicate statistically significant correlations (p < 0.05).

## 4 Discussion

MASLD is an increasingly common obesity-related complication in children; however, early non-invasive biomarkers remain limited. Given the regulatory role of exosomal miRNAs in metabolic and hepatic processes, this study aimed to identify MASLD-specific miRNAs in children with obesity and assess their association with liver function and insulin resistance indices. Among the six differentially expressed miRNAs identified, miR-122-5p, miR-34a-5p, and miR-885-5p were significantly upregulated, whereas miR-32-3p and miR-570-3p were downregulated in children with MASLD. Notably, these miRNAs showed significant correlations with clinical parameters, such as ALT, and uric acid levels and TyG-based insulin resistance indices, suggesting their involvement in hepatic injury and metabolic dysregulation. These findings highlight the potential utility of exosomal miRNAs as early indicators of MASLD and provide insights into their possible pathophysiological roles in pediatric liver disease. However, given the small sample size (n=30), the statistical power of the study is limited, and the results should be considered exploratory and hypothesis-generating.

Among the miRNAs upregulated in the children with both obesity and MASLD, miR-34a-5p, miR-122-5p, miR-885-5p, and miR-885-3p were significantly associated with markers of liver damage and insulin resistance. These miRNAs have been linked to various pathological processes in the liver, including fibrosis, inflammation, and lipid accumulation. Notably, previous studies have established strong associations of miR-34a and miR-122 with liver injury and disease severity in adults with MASLD and other liver diseases (14–20). Its elevated expression in our study suggests a comparable pathological role in pediatric MASLD, potentially contributing to liver damage and insulin resistance in children with obesity and MASLD. The upregulation of miR-885-5p and miR-885-3p, although less studied than that of miR-34a and miR-122, may also be relevant to the pathogenesis of pediatric MASLD. These miRNAs are implicated in cellular stress responses and lipid metabolism, which are crucial for MASLD progression. Their upregulation in the children with both obesity and MASLD could indicate a response to metabolic stress and liver inflammation, potentially exacerbating liver damage and promoting insulin resistance. This suggests that miR-885-5p and miR-885-3p could serve as novel biomarkers or therapeutic targets for the management of MASLD in children with obesity.

Conversely, miR-32-3p and miR-570-3p were downregulated in the children with both obesity and MASLD, indicating their protective role against liver damage. The downregulation of these miRNAs in MASLD suggests a potential loss of protective mechanisms that mitigate liver damage and inflammation. miR-32-3p regulates cellular apoptosis and inflammation, processes relevant to liver health. Its low expression in children with MASLD may reduce its protective effects, thereby contributing to a more severe disease state. Similarly, miR-570-3p downregulation may indicate a reduced ability to counteract liver damage, as this miRNA is thought to play a role in inflammation and immune response (21, 22). The reduction in these miRNAs may reflect a failure to effectively modulate liver injury, allowing for the progression of MASLD in the presence of obesity.

Our findings are consistent with those of previous studies that reported the upregulation of miR-122-5p and miR-34a-5p in pediatric MASLD. Brandt et al. demonstrated elevated circulating levels of miR-122 in prepubertal children with obesity and MASLD, along with positive correlations with ALT and CK18 levels, underscoring its hepatocyte-specific origin and diagnostic potential (23). Similarly, Zhou et al. identified increased expression of exosomal miR-122-5p and miR-34a-5p in Chinese children with MASLD and reported their association with transaminases and uric acid (24). However, unlike Zhou’s study, which only compared patients with MASLD with healthy controls, our study included an additional group of children with obesity without MASLD, allowing for a more disease-specific evaluation of miRNA signatures in children with obesity. Furthermore, Lin et al. examined circulating miRNAs in adolescents with hepatosteatosis and insulin resistance and reported elevated miR-155-5p levels in those with coexisting MASLD and IR, but not in those with MASLD alone (12). In contrast, our study demonstrated that miR-122-5p, miR-34a-5p, and miR-885-5p were specifically upregulated even in the absence of IR. These findings may suggest a potential involvement of these miRNAs in early hepatic alterations, although this should be interpreted with caution given the small cross-sectional sample size. Using liver biopsies, Li et al. confirmed MASLD in adolescents and identified miR-122-5p and miR-193a-5p as key plasma biomarkers associated with histological severity (25), supporting the hepatic specificity of these molecules across age groups and diagnostic modalities. Lastly, Behrooz et al. reported elevated miR-122 levels in peripheral blood mononuclear cells of children with obesity and their correlation with triglycerides and inflammatory markers (26). However, their study did not assess MASLD status, which limits disease specificity. By focusing on exosomal miRNAs that are likely derived from hepatocytes, our study offers a more targeted approach for detecting MASLD -related molecular alterations in children with obesity.

This study had several notable strengths. First, by including an intermediate comparison group of children with obesity without MASLD, we were able to delineate miRNA expression changes specific to MASLD rather than to obesity per se. This design provides strong evidence for the disease specificity of the identified miRNA biomarkers. Second, the use of exosome-derived miRNAs rather than total circulating or cellular miRNAs enhances the likelihood that the observed signals are of hepatocellular origin, given that exosomal cargo is known to mirror the physiological state of source tissues. Third, our focus on prepubertal children minimized the potential confounding effects of pubertal hormones on insulin sensitivity and liver metabolism, making our findings more attributable to early metabolic alterations associated with MASLD.

This study had some limitations. Its cross-sectional design precludes causal inferences, and the small sample size may limit its generalizability. MASLD diagnosis was based on ultrasound rather than biopsy, which restricts histological precision. Furthermore, the functional roles of the identified miRNAs remain unclear.

Future studies should validate these findings in larger longitudinal cohorts and investigate the mechanistic roles of the candidate miRNAs in hepatic metabolism. Exosomal miRNA profiling may ultimately contribute to the early, non-invasive diagnosis and personalized risk assessment of pediatric MASLD.

## 5 Conclusions

Specific miRNAs, particularly 34a-5p, 122-5p, 885-5p, 885-3p, 570-3p, and 32-3p, were differentially expressed in children with MASLD compared with children with obesity and healthy controls. These miRNAs were closely associated with liver function and insulin resistance, suggesting their potential as biomarkers for MASLD and related metabolic disturbances in pediatric patients.

## Data Availability

The data analyzed in this study is subject to the following licenses/restrictions: Data are not publicly available due to ethical restrictions involving patient confidentiality. Requests to access these datasets should be directed to bbananet1@naver.com.
